# FRIENDS WITH UNEXPECTED BENEFITS: COACHING CAREGIVERS OF PEOPLE LIVING WITH DEMENTIA

**DOI:** 10.1093/geroni/igae098.1764

**Published:** 2024-12-31

**Authors:** Rita Jablonski, Amy Cleverdon, Chunhong Xiao, Reed Bratches

**Affiliations:** University of Alabama at Birmingham, Birmingham, Alabama, United States; University of Alabama at Birmingham, Birmingham, Alabama, United States; University of Alabama at Birmingham/School of Nursing, Birmingham, Alabama, United States; University of Alabama at Birmingham, Birmingham, Alabama, United States

## Abstract

Persons living with dementia (PLWD) often exhibit care-resistant behaviors (CRBs), including refusing to take medications or bathe. This randomized clinical trial is currently enrolling caregivers of people living with dementia, who receive nine one-hour coaching sessions designed to help them prevent and manage CRBs. Coaching is the application of evidence-based strategies delivered in a conversational manner; it is a bidirectional, problem-solving approach designed to improve the ability of family caregivers to recognize, prevent, and manage CRB in their daily interactions with their care recipients. We report qualitative findings from 13 caregivers who completed all coaching sessions. The results of the thematic analysis revealed unexpected benefits and feedback. Participants reported a renewed sense of hope and optimism “after nearly giving up.” They expressed feelings of relief that the dementia-related brain changes were responsible for behaviors, not “caregiver mistakes,” or “they are doing this on purpose,” which led to greater compassion for their care recipients. These new insights also contributed to a permanent adjustment of their expectations of their care recipients’ cognition, and re-established connections with PLWD. The participants applied newly learned skills and strategies in unexpected situations, such as improving cooperation during travel and medical appointments. Participants also shared that they taught secondary caregivers, such as sitters, specific techniques to maintain continuity of care. These findings support the need for future studies that emphasize targeted, personalized interactions inherent in this coaching intervention, versus passive presentation of generic information.

